# Interplay Between Immune Microenvironment CD8^+^ Tumor-Infiltrating Lymphocytes and PDL-1 Expression as Prognostic Markers in Invasive Cervical Squamous Cell Carcinoma

**DOI:** 10.3390/medicina61112007

**Published:** 2025-11-10

**Authors:** Laura-Andra Petrică, Mariana Deacu, Georgeta Camelia Cozaru, Anca Florentina Mitroi, Gabriela Izabela Bălţătescu, Manuela Enciu, Oana Cojocaru, Anca-Antonela Nicolau, Andrei Radu Baz, Lucian Șerbănescu, Mariana Aşchie

**Affiliations:** 1 “St. Apostol Andrew” Emergency County Hospital, 145 Tomis Blvd., 900591 Constanta, Romania; 2 Faculty of Medicine, Ovidius University of Constanta, 1 Universitatii Alley, 900470 Constanta, Romania; 3 Research Center for the Morphological and Genetic Study in Malignant Pathology (CEDMOG), Ovidius University of Constanța, 145 Tomis Avenue, 900591 Constanta, Romania; 4 Academy of Romanian Scientists, 3 Ilfov Street, 050045 Bucharest, Romania; 5 Institute of Doctoral Studies, Ovidius University of Constanta, 1 Universitatii Street, 900470 Constanta, Romania

**Keywords:** invasive squamous cell carcinoma, cervix, tumor microenvironment, CD8^+^ tumor-infiltrating lymphocytes, PD-L1expression, Ki-67 expression

## Abstract

*Background*: Cervical cancer remains a major cause of cancer-related morbidity and mortality worldwide, with limited therapeutic options for advanced disease. As we better understand the fine mechanisms involved in the interaction between tumor cells and the tumor microenvironment, new paths and opportunities will emerge. Recent evidence highlights the prognostic and predictive roles of immune checkpoint markers and tumor-infiltrating lymphocytes (TILs), especially CD8^+^ TILs, in shaping treatment outcomes. *Objectives*: This study investigated the immunohistochemical expression of PD-L1 and CD8^+^ TILs in 48 newly diagnosed, treatment-naive cervical cancer cases and analyzed their associations with clinicopathological features and survival outcomes. *Results*: In our cohort, we observed that PD-L1 positivity was identified in 68.8% of cases, most frequently in advanced FIGO stages and in tumors with lympho-vascular invasion or with a high proliferation rate evaluated by the Ki-67 index. High levels of intra-tumoral CD8^+^ TILs were observed in 52.1% of cases and correlated positively with stromal TILs, lower proliferation rates, and absence of vascular invasion. A significant inverse relationship was found between PD-L1 expression and the density of CD8^+^ TILs (*p* = 0.047). Survival analysis showed that patients exhibiting a “cold” immunophenotype with low levels of CD8^+^ TILs and PD-L1-positive tumors had worse outcomes, while high levels of CD8^+^ TILs played a protective role. *Conclusions*: Our study highlights the importance of the immunohistochemical assessment of PD-L1 and CD8^+^ TILs biomarkers, which have a complementary inter-relationship and have a significant prognostic impact on cervical squamous cell carcinoma. PD-L1 positivity marks aggressive disease features, while higher intra-tumoral CD8^+^ TIL density is protective. Their combined evaluation may improve patient stratification and inform immunotherapy strategies.

## 1. Introduction

Carcinoma of the cervix continues to be one of the most important causes of morbidity and mortality for women worldwide with 600,000 new cases (~6.5% of all cancers) and 342,000 deaths (7.5% of cancer deaths) recorded globally in 2020, particularly affecting low-resource countries where advanced-stage diagnosis is common [1,2]. While preventive strategies, such as HPV (human papillomavirus) vaccination, have reduced incidence in some regions, treatment options for advanced disease remain limited, and resistance to standard therapies poses an ongoing clinical challenge. Currently, these patients benefit from chemotherapy, radiotherapy, and surgery, but sometimes these are ineffective, making the search for new therapeutic options an important subject for future research [3].

An assessment of the immune system’s function is a developing topic in oncological research. Studies performed during the last two decades have proved that the immune response of the host plays a key role in evolution of cancer. Immunotherapy has shown positive results and is considered a useful therapeutic tool for many types of cancer, such as malignant melanoma, lung, and invasive urothelial carcinoma [4]. This area of research has the capacity to uncover novel therapeutic targets for cervical cancer as well. These new findings require increased attention in the analysis of the tumor microenvironment regarding the composition of tumor-infiltrating lymphocytes (TILs) and their interaction with tumor cells.

Recent studies indicate that one of the most important contributing factors to anti-tumor immune response is tumor-infiltrating lymphocytes positive for the CD8 marker (CD8^+^ TILs), a marker of cytotoxic-T cells [5]. The presence of CD8^+^ TILs, along with other immune cells, has the potential to improve outcomes and patient prognosis [6,7,8]. The primary function of CD8^+^ TILs is to identify neoplastic antigens and subsequently activate the immune system; however, they can be affected by several mechanisms that alter their function and effectiveness. Many studies have highlighted the role of Programmed Death-1 (PD-1) and its ligand, Programmed Death Ligand 1 (PD-L1), on the anti-tumor activity of TILs. PD-1 is usually expressed by CD8^+^ T-lymphocytes and, when bound with PD-L1, expressed mainly by tumor cells, acts like a brake to the cell-mediated immune responses of T lymphocytes [5]. For this reason, PD-L1 overexpression is associated with poor prognosis in many types of cancer and checkpoint blockade has improved outcomes in selected cancers [9,10,11].

Although PD-L1 expression is a biomarker clinically approved for immunotherapy in metastatic or recurrent cervical cancer (based on the KEYNOTE-158 and -826 trials), its prognostic significance in treatment-naive invasive squamous cell carcinoma (SCC) remains unclear [12,13]. Furthermore, the relationship between PD-L1 expression and CD8^+^ TILs density—especially their combined prognostic value—has not been adequately explored in newly diagnosed cases.

Currently, recommendations for immunotherapy in newly diagnosed cervical cancer are still under debate, and there is limited evidence to support its benefit for these patients, particularly in cases presenting with advanced-stage disease at the time of diagnosis. In the latter scenario, therapeutic options are limited to palliative solutions.

The main goal of our retrospective–prospective study is to analyze the immunohistochemical expression of PD-L1 alongside CD8-positive tumor-infiltrating lymphocytes in newly diagnosed patients with cervical invasive squamous cell carcinoma without prior neoadjuvant therapy and to establish their prognostic role. This combined analysis of PD-L1 and CD8^+^ TILs status can provide meaningful prognostic insights and may help identify patients who could benefit from immune-based stratification, even before systemic therapy is initiated. In addition, we examine their correlations with the most significant clinical, morpho-pathological parameters, as well as prognostic immunohistochemical markers, thereby aiming to contribute valuable insights to the field of oncology and enhance understanding of tumor characteristics and microenvironment.

## 2. Materials and Methods

### 2.1. Data and Specimen Selection

This retrospective–prospective study was conducted in the St. Andrew’s Clinical Emergency County Hospital of Constanta, Romania, during 2019–2022. We extracted the cases with anatomopathological diagnosis of invasive squamous carcinoma of the cervix. The exclusion criteria included cases with anatomopathological diagnoses made outside the “St. Andrew” Constanța County Emergency Clinical Hospital, as well as cases with benign cervical lesions. Additionally, tumors of non-epithelial origin, secondary (metastatic) cervical tumors, recurring disease, and cases with insufficient biological material or extensive tumor necrosis were excluded from the study. From 179 cases, we selected 48 cases with pathological diagnosis of invasive squamous cell carcinoma of the cervix, the majority of which were based on biopsy (46 cases) or simple hysterectomy specimens, which were confirmed in the Clinical Service of Pathology Department, from women with no prior neoadjuvant chemotherapy or radiotherapy. This retrospective–prospective study was approved by the Institutional Ethics Review Board (ID: 42006) and conducted following the Declaration of Helsinki and Good Clinical Practice Guidelines. Clinico-pathological features were extracted from the electronic database and from the pathology reports of the hospital. We obtained medical history; complete blood counts from the laboratory department; the serum creatinine level to explore kidney function; and imaging investigations, like computed tomography (CT), magnetic resonance imaging (MRI), or abdominal echography. Patients received treatment that consisted of radical hysterectomy and/or chemotherapy and/or radio/brachytherapy according to the national medical guidelines for their clinical stage and biological status. Follow-up clinical data for each patient was extracted from electronic hospital records or the patient’s contact.

### 2.2. Morfopathological Evaluation

The slides of the cases included in the study were stained with hematoxylin–eosin (HE) and re-evaluated blindly and independently by two pathologists for diagnostic confirmation to check the quality of the biological material. The immune response of the host was analyzed through characterization of tumor stromal-infiltrating lymphocytes (TILs) on HE slides. Stromal TIL selection and scoring were performed on digitized whole-slide images of H&E-stained sections, following International Immuno-Oncology Biomarkers Working Group recommendations. Only stromal areas within the invasive tumor were assessed; necrosis, ulceration, cautery or crush artifact, intraepithelial lesions, and peritumoral stroma were excluded. Two pathologists, blinded to clinicopathologic data, independently estimated the stromal TIL percentage. According to the guidelines of Hendry S. et. al. (2017), patients were divided into three groups using the following cut-off values: <10% (low level of TILs), 10–40% (moderate level), and >40% (high level) [14]. When the two assessments did not agree, a third pathologist reviewed the same whole-slide images and issued the final score. No formal interobserver concordance analysis was performed.

### 2.3. Immunohistochemical Evaluation

Immunohistochemistry (IHC) was performed using the following antibodies and methods of quantification:

1. PD-L1 with CAL10 clone, a mouse monoclonal antibody from Master Diagnostica, Spain; dilution 1:50; antigen retrieval was performed by heat-induced epitope retrieval (HIER) using citrate buffer (pH 6.0) at 98 °C for 20 min; a positive control was performed on placenta tissue. The quantification of this biomarker was carried out following recommendations from the latest clinical trials, including the presence of at least 100 viable tumor cells in the section. Both tumor cells and inflammatory cells were taken into consideration using a high-power microscopic field. A positive reaction for tumor cells was considered when partial or complete membranous staining (of any intensity), distinct from cytoplasmic staining, was observed, and immune cells were considered positive for any staining. A combined positive score (CPS) was used to assess PD-L1 expression, defined as the number of PD-L1-positive cells (tumor cells, lymphocytes and macrophages) divided by the total number of tumor cells (positive + negative) × 100 [15], with a maximum score defined as a CPS of 100. According to Monsrud AL et al. (2023), a 10% cut-off value has a statistically significant impact on prognosis for newly diagnosed patients, and we adopted this cut-off value into our methodology, dividing cases into negative PD-L1 (<10%) and positive PD-L1 (≥10%) [16].
2. CD8 (C8/144B clone with mouse monoclonal antibody “ready to use” from Biocare, U.S.A.; antigen retrieval was performed using heat-induced epitope retrieval (HIER) in Tris-EDTA buffer (pH 9.0) at 98 °C for 20 min; a membranous/cytoplasmic-positive immunoreaction and positive control were performed on tonsil tissue). A positive reaction was considered when at least one cell was immunostain [6]. By immunohistochemistry, CD8^+^ TILs density was quantified exclusively within the intra-tumoral compartment (tumor nests), excluding the peritumoral stroma.
3. Quantification was performed using whole-slide digital images and reviewed using specific software. Areas of maximal CD8^+^ infiltration (“hot spots”) were selected for each case. From these regions, five random high-power fields (HPFs) at 400× magnification were evaluated, and CD8^+^ positive cells were manually counted in each HPF. Cells showing clear membranous or cytoplasmic staining were considered positive. The mean number of CD8^+^ TILs per HPF was calculated for each case, and the median value across the cohort was used as a threshold to stratify patients into low and high CD8^+^ TILs groups.
4. Ki-67- SP6 clone with rabbit monoclonal antibody—“ready to use” from BioCare, U.S.A.; antigen retrieval was performed by heat-induced epitope retrieval (HIER) using citrate buffer (pH 6.0) at 98 °C for 20 min; a nuclear-positive immunoreaction and positive control were performed on tonsil tissue. The nuclear proliferation rate was obtained by counting positive nuclear cells from 500 cells [17], and the scores were stratified into 3 groups: values lower than 30%—low proliferation group; values between 30% and 50%- medium proliferation group; values higher than 50%- high proliferation group [18]. We also stratified Ki-67 based on the cut-off value of 42% in the low category (<42%) and high category (>42%) since the study by Tu Y. et al. (2022) proved that this value has prognostic value in cervical cancer [19].

### 2.4. Statistical Analysis

Statistical processing was performed for the collected data using IBM SPSS Statistics software, version 26 (IBM Corp., Armonk, NY, USA). Descriptive statistics summarize continuous data using mean, median, standard deviation (SD) and interquartile range, and categorical data as counts and percentages. For categorical associations, we used Pearson’s chi-square test or Fisher’s exact test when expected counts were <5; for ordered categories we used the chi-square test for trend (linear-by-linear association). For non-normally distributed continuous variables, two-group comparisons used the Mann–Whitney U test and >2 groups used the Kruskal–Wallis test. Associations between continuous or ordinal variables were assessed with Spearman’s rank correlation coefficient (ρ).

Overall survival (OS) was determined as the duration from the point of diagnosis until death or the latest follow-up interaction (censored). Time-to-event distributions were estimated with Kaplan–Meier curves and compared using the log-rank test. Variables significant in univariable analyses were entered into multivariable Cox proportional hazards regression.

A statistical test is considered significant when the *p*-value is less than 0.05 (2-tailed).

## 3. Results

The median age of the women included in our study was 56.5 (32–83) years old, 66.7% of whom were 66.7 years old and mostly from urban areas (52.1%). The main clinical symptoms that patients reported to the physician were vaginal bleeding or pelvic-abdominal pain. Following a clinical and imaging evaluation, a cervical tumor was identified, frequently FIGO stage IIB (22.9%), followed by FIGO stage IIIB (16.7%) and IIIC (14.6%). In 72.9% of cases, unilateral or bilateral parametrial invasion was observed.

The records for complete blood counts were available in 47 cases, and the following results were extracted: leukocytes/microL with a mean (SD) of 10,610.6 ± 3.943, with 47.9% of cases having counts less than 10,000/microL.

Regarding morpho-pathological aspects, 56.3% of cases were diagnosed with non-keratinized squamous cell carcinoma, followed by the keratinized squamous cell carcinoma subtype (31.3%). Poorly differentiated cervical carcinoma (G3) was most frequent, presenting in 35 cases (72.9%); the presence of squamous intraepithelial neoplasia (SIL)/cervical intraepithelial neoplasia (CIN) lesions was identified at a rate of 39.6%, the majority of which were high grade. Lympho-vascular invasion was identified in 62.5% of cases. A high nuclear proliferation rate (index Ki-67 > 50%) was recorded in 43.8% of cases; using the 42% cut-off value, we observed 30 cases with an index of Ki-67 greater than 42%.

The assessment of the inflammatory microenvironment revealed the presence of TILs in all cases: 23 cases had high-level TILs and 52.1% of cases had a high density of CD8^+^ TILs (HD-CD8^+^ TILs). No significant differences were found between leukocyte counts across the three stromal TIL categories (H (2) = 0.605, *p* = 0.739) or CD8^+^ TIL levels (U = 242.00; *p* = 0,482).

Low density levels of CD8^+^ TILs (LD-CD8^+^ TILs) were identified in 23 cases, which mostly comprised patients (60.9%) over 56.5 years old, in contrast to HD-CD8^+^ TILs frequently encountered in patients under the median age of our cohort (60.0%). For both categories of CD8-TILs, most patients were between 45 and 69 years old without a statistically significant difference (*p* = 0.065). In LD-CD8^+^ TILs, 54.5% of cases had a leukocyte blood count less than 10,000/microL, and in high-CD8^+^ TILs cases, 56.0% of cases had a leukocyte blood count greater than 10,000/microL, without a statistically significant difference (*p* = 0.564). In this cohort of patients, CD8^+^ TILs levels did not differ by hemoglobin (<12 g/dL/≥12 g/dL), rural versus urban areas (*p* = 0.571), tumoral size (<4 cm/≥4 cm), the presence or absence of metastases on lymph nodes or parametrial invasion or grade (moderate versus poorly differentiated tumors; *p* = 0.424). Significant associations were observed between levels of CD8^+^ TILs and TILs category (*p* < 0.001), the absence or presence of lympho-vascular invasion (*p* = 0.008), and Ki-67index (<42% vs. ≥42%; *p* = 0.031).

**Figure 1:**
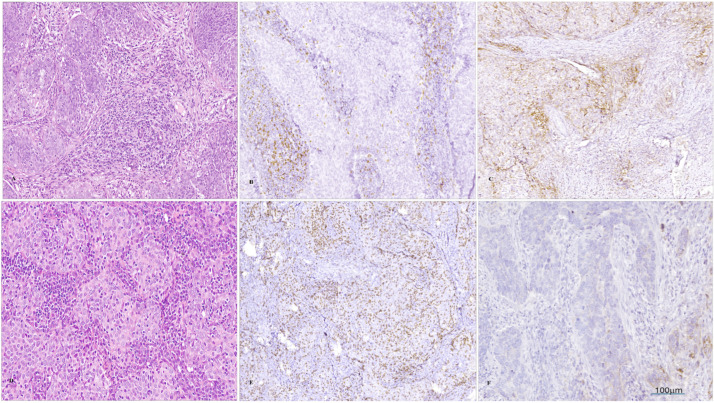
Microscopical images of a case where invasive SCC was diagnosed ((**A**), col Hematoxylin -eosin, magnification 100×) with low levels of intratumoral CD8^+^ TILs ((**B**); IHC, magnification 100×) and positive PD-L1 ((**C**); IHC, magnification 100×); another case was diagnosed with invasive SCC ((**D**), col Hematoxylin–eosin, magnification 100×), with high levels of intratumoral CD8^+^ TILs ((**E**); IHC, ob. 100×) and negative PD-L1 ((**F**); IHC, magnification 100×).

**Figure 2:**
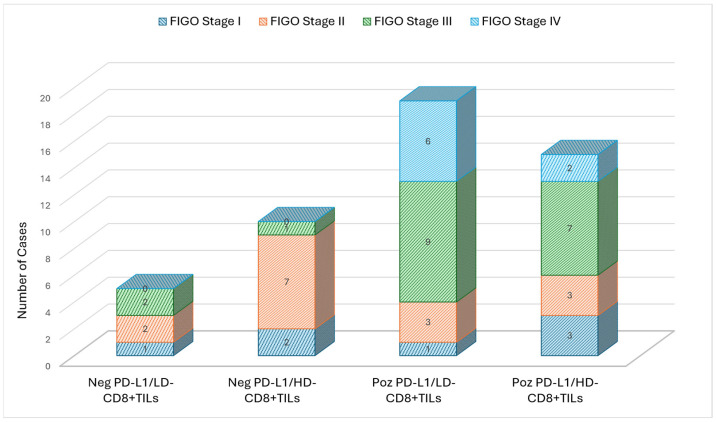
Case distribution according to clinical FIGO stage and expression of PD-L1 and CD8^+^ TILs.

PD-L1 positivity was obtained in 33 cases (68.8%), and this was recorded more frequently in patients with FIGO stage III (48.5%) and IV (24.2%) compared to cases with an early clinical stage (Figure 1). We also noticed that PD-L1-positive cells were present more frequently (74.3%) in cases with a poor differentiation grade, but the results were not statistically significant. All cases with low levels of TILs (<10%) were PD-L1-positive, and 86.7% of PD-L1-negative cases had higher levels of TILs (≥40%). These results were statistically significant (*p* < 0.001) but showed a negative correlation coefficient (ρ = −0.526, *p* < 0.001). A similar result was obtained when analyzing differences between PD-L1 expressions and levels of CD8^+^ TILs: 73.3% of cases in the negative PD-L1 group showed high levels of CD8^+^ TILs, and 57.6% of cases with positive PD-L1 showed low levels of CD8^+^ TILs. The latter was also characterized by the highest frequency (*n*-9) of FIGO stage IV cases (Figure 2). These differences proved to be statistically significant (*p* = 0.047) and negatively correlated (ρ = −0.287, *p* = 0.048). We also noticed statistically significant differences between PD-L1 expressions and invasion of the parametrium, with lympho-vascular invasion, lymph node metastases, and the Ki-67 index (Table 1).

**Figure 3:**
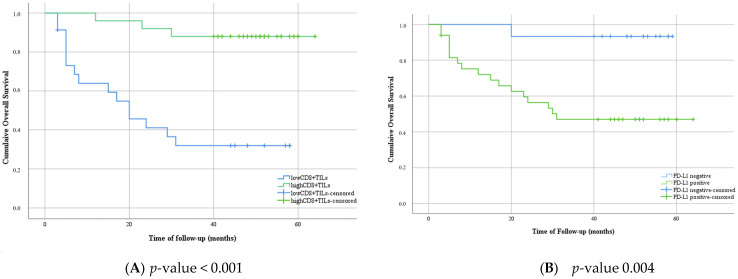

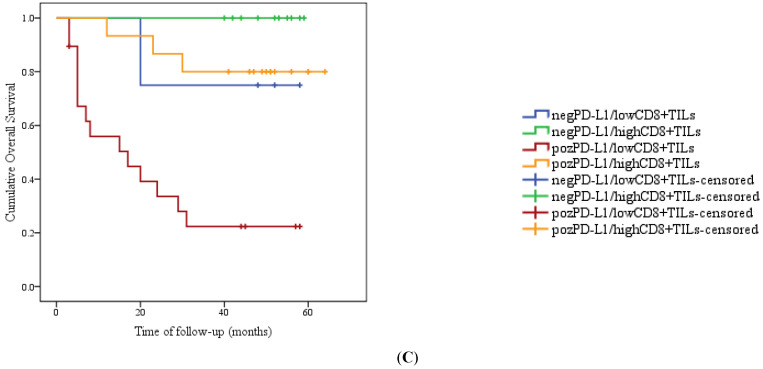
Kaplan–Meier plot of overall survival in categories with low or high levels of CD8^+^ TILs (**A**); negative expression versus positive expression of PD-L1 (**B**), and different groups based on the immunostaining evaluation of PD-L1 and CD8^+^ TILs (**C**).

The follow-up ranged from 3 months to 64 months, with a median of 44.5 months. During the follow-up period, 18 patients died from cervical cancer, and 2 patients were lost to follow-up. Survival analysis proved that patients with positive PD-L1 presented with shortened survival compared with PD-L1-negative patients (Figure 3A). Inversely, patients who exhibited higher levels of CD8^+^ TILs presented a lower mortality risk than patients with low levels of CD8^+^ TILS (Figure 3B). All patients with negative PD-L1 and high levels of CD8^+^ TILs were still alive at the end of the follow-up period (Figure 3C). Multivariate analysis of overall survival was conducted for several important factors; lymph node metastases were statistically significant, followed by CD8^+^ TILs levels (Table 2).

## 4. Discussion

Cervical cancer is the fourth most common cancer among women worldwide [20]. One of the most important causes identified is persistent infection with high-risk types of human papillomavirus (HPV) [20], but other factors are also involved. Discoveries are being made as our knowledge regarding this type of cancer is evolving. It represents a significant global public health issue with the highest rates reported in low-income and developing countries, mostly due to inefficient or non-existent cervical cancer screening and vaccination programs [21]. Advanced stages of cervical cancer are more frequently diagnosed in disadvantaged regions, which significantly limits curative treatment options, sometimes limited to purely palliative care. Also, patients who are diagnosed in the early stages can develop resistance or experience disease recurrence. The study by Talib WH et al. (2021) noted that half of patients treated with chemotherapy develop resistance, which further complicates disease management [22].

Squamous cell carcinoma, followed by adenocarcinoma, are the most common morphological types of cervical carcinoma [23]. These two forms differ both in terms of oncogenic mutations [23] and the specific immunological features of the tumor microenvironment [24]; for this reason, we did not include this morphological type of cervical cancer in our research. Unfortunately, the number of patients with primary cervical adenocarcinoma is increasing, associated with a lower survival rate compared to those with squamous cell carcinoma, especially when metastases in regional lymph nodes are present [25,26]. Despite these differences, the therapeutic approach is the same for both types of cervical cancer; future studies must address different forms of clinical management based on cervical cancer’s morphology [27].

As our understanding of the intricate mechanisms underlying the interplay between neoplastic cells and the tumor microenvironment deepens, new prospects will arise for the formulation of groundbreaking, tailored pharmacological molecules adept at enhancing therapeutic outcomes, particularly for individuals afflicted with advanced disease stages. Over the past two decades, remarkable discoveries have led to a better understanding of the immune microenvironment’s complexity, enhancing immunotherapeutic strategies for cervical cancer and the development of effective personalized treatments. These insights tackle the multiple problems and challenges associated with disease progression and have the potential to improve survival in advanced disease.

Malignant cells grow, metastasize, and escape immune surveillance within a permissive tumor microenvironment (TME). TME is extremely complex and comprises stromal cells, including fibroblasts, vascular endothelial cells, extracellular matrix components, different types of inflammatory cells, and several biomolecules (growth factors, cytokines, chemokines), which are finely integrated into a complex system that can either aid or block tumoral progression [27].

A major research focus is the immune components of the TME [28]. In the presence of malignant cells, immune “tumor-associated” cells or tumor-infiltrating lymphocytes (TILs) can either promote tumor progression or enhance anti-tumor immunity, depending on their functional state and interaction within the tumor microenvironment [29]. The study by Labani MA et al. (2020) describes three stages regarding the interaction between tumor cells and immune cells: firstly, the immune cells identify and destroy most tumor cells [30]. This is termed the “elimination phase”. Some tumor cells have the property to “hide” from the immune system and, consequently, contribute to tumor development. This marks the onset of the “equilibrium phase” [30]. As neoplastic cells and their surrounding stromal environment progress, the mechanisms of immunosuppression become more pronounced, ultimately leading to the “escape phase” [30]. Understanding this dynamic is crucial for developing effective, patient-tailored immunotherapies.

Several studies link TILs to prognosis in many types of cancer, including cervical cancer [31]. Wild CM et al. (2023), incorporating 238 types of cervical cancer with different morphological subtypes, as well as invasive SCC and adenocarcinoma, demonstrate that the presence of TILs can be an important and independently favorable prognostic factor [31]. It was also observed that cervical cancer usually has a strong immune response, and positive responses after immunotherapy can be significant in selected patients [32]. In the present study, we also observed that most patients (47.9%) had higher levels of TILs (more than 40%), and these values were associated with a better overall outcome. D’Alessandris N. et al. (2021) similarly observed higher levels of TILs in patients with complete pathological response, reinforcing the supposition that immunotherapy has an important role in outcome improvement [32]. We also investigated whether the levels of TILs present in the tumoral stroma are correlated with the levels of leukocytes from the peripheral blood. Even if we recorded higher levels of leukocytes in patients with higher TILs, no significant difference was demonstrated. This may indicate that local immune infiltration is largely independent of the systemic inflammatory milieu as measured by routine hematologic indices, reinforcing the role of TILs as a potentially independent prognostic marker.

Several types of immune cells were identified, each with a different role in defense mechanisms: immune cytotoxic CD8^+^ T cells, CD4^+^ T cells, natural killer cells (NK cells), B lymphocytes, dendritic cells, tumor-associated macrophages (TAMs), myeloid-derived suppressor cells (MDSCs), and regulatory T cells (Treg cells) [27]. Most immune cells are tumor-infiltrating lymphocytes (TILs), mainly T lymphocytes, from which CD8^+^ T lymphocytes play a pivotal role in the immune landscape of cervical cancer [27]. CD4^+^ T lymphocytes are also important as they are involved in the activation and regulation of CD8^+^ T lymphocyte responses in the tumor microenvironment [29].

Different studies describe how the presence of CD8^+^ TILs is associated with improved prognosis in cervical cancer patients, highlighting their potential as biomarkers for therapeutic response and patient survival [29,31]. Previously, researchers assessed the levels of CD8^+^ TILs in both intra-tumoral and peripheral compartments of the malignant tumor, but the systematic research conducted by Tang et al. (2021) demonstrated that only intratumoral CD8^+^ TILs are associated with improving disease-free and overall survival [33]. Considering these results, we only quantified intratumoral CD8^+^ TILs and we recorded a rate 52.1% with high densities of intratumoral CD8^+^ TILs in our cohort. We also observed a strong and positive correlation between the levels of CD8^+^ TILs and the density of TILs categories, with better outcomes for those patients with high levels of CD8^+^ TILs.

Our study did not identify any association between levels of CD8^+^ TILs and clinical factors like age or peripheral leukocytes indicating that CD8^+^ TILs status is specific to the microenvironment of tumor cells. In addition, we noticed that there are statistically significant differences in morphological features usually associated with tumor aggressiveness, such as the presence of lympho-vascular invasion or a high proliferation rate. We observed that high CD8^+^ TIL levels correlated positively with lower Ki-67 proliferation index (*p* = 0.031) and with the absence of lymphovascular invasion (*p* = 0.008)—two pathological features associated with less aggressive tumor behavior, suggesting that levels of CD8^+^ TILs are influenced by tumor biology. This finding reinforces the notion that a robust immune response within the tumor microenvironment may suppress tumor progression and constrain its invasive potential, even prior to therapeutic intervention.

Further investigation into the role of CD8^+^ T lymphocytes may enhance our understanding of cervical cancer immunology and lead to improved therapeutic strategies tailored to individual patient profiles. CD8^+^ cytotoxic T lymphocytes are usually found in cervical cancer tissue, but their presence and density can vary significantly, influencing the overall immune response. Moreover, their function can be compromised due to various immunosuppressive mechanisms mediated by both tumor cells and associated stroma cells. HPV infection has a major impact on the function of CD8^+^ T lymphocytes as it can induce a state of “exhaustion” in these lymphocytes, resulting in the failure to successfully eliminate HPV-infected cervical cells or induce a downregulation of MHC class I antigen presentation, thereby impairing the recognition of tumor cells by the cytolytic CD8^+^ lymphocytes [34]. However, one of the most important anti-tumor mechanisms, which affects the normal activity of TILs and also leads to the abnormal function of CD8^+^ TILs, is represented by the programmed Death-1 (PD-1) and its ligand, the Programmed Death Ligand 1 (PD-L1) axis, which are key coinhibitory immune-checkpoint regulators and deliver inhibitory signals that constrain T-cell activation, proliferation, and effector function, thereby maintaining peripheral tolerance and shaping anti-tumor immunity [34].

An immunohistochemical assessment for PD-L1 is increasingly important in many types of cancer, with well-established cut-off values, because immunotherapy recommendations are based on its correct evaluation. This therapeutic option is currently approved by the FDA for locally advanced or metastatic cervical cancer and large clinical trials like KEYNOTE-158 and KEYNOTE-826 158 [12,13]. In these studies, it was proven that immunotherapy with Pembrolizumab (Keytruda) has therapeutic benefits for patients with recurrent, persistent, and metastatic disease after chemotherapy. In these clinical studies, two cut-off values were used for PD-L1 positivity: ≥1% and ≥10% [12,13]. A higher response rate was observed in patients with over 10% PD-L1-positive cells [35].

For newly diagnosed patients with cervical cancer, without previous treatment, few studies have assessed a cut-off value. While the KEYNOTE-158 and KEYNOTE-826 clinical trials used a CPS ≥ 1 as the therapeutic threshold for pembrolizumab eligibility in cervical cancer, we selected a higher cut-off (CPS ≥ 10) for this study based on evidence from Monsrud et al. (2023), which demonstrated that this threshold better stratified patients according to survival outcomes in treatment-naïve cervical squamous cell carcinoma [16].

The study by Monsrud et al. (2023), based on a cohort of 73 cases, proved that a cut-off value ≥10% for PD-L1 has prognostic value compared to a cut-off value ≥1% [16]. Our aim was to evaluate PD-L1 primarily as a prognostic biomarker rather than a predictive marker of immunotherapy response. For this reason, we adopted this cut-off value in our methodology of quantification and recorded 68.8% cases with positive PD-L1, which is in the range of 19% to 88% reported in academic literature [7,16]. Most PD-L1-positive cases were high grade (78.8%). Variations in positive PD-L1 can be attributed to various factors, including the specific antibody used for assessment, staining techniques, and scoring algorithms; assessments can be improved using accepted scoring methods, such as the combined positive score (CPS) [16].

Several studies observed a close connection between PD-L1 expression and levels of CD8^+^ TILs. The study by Ishikawa M. et al. (2020) and Chen J et al. (2020) proved that PD-L1 expression is negatively correlated with the levels of CD8^+^ TILS [36,37]. Our research recorded similar results and is in agreement with this observation regarding prognosis: patients with a PD-L1-positive expression and low levels of CD8^+^ TILs have worse overall survival compared to cases in which PD-L1 is negative but higher levels of CD8^+^ TILs are present [16,37].

The finding that in our study all cases with low stromal TILs (<10%) were PD-L1 positive may reflect both spatial immune heterogeneity and a distinct biological phenotype. From a technical standpoint, uneven intratumoral distribution of immune cells—commonly observed in cervical cancer—can lead to underrepresentation of TILs in small or superficial biopsies. This spatial variability has been demonstrated in spatial transcriptomic and multiplex IHC studies, where immune cells cluster in stromal zones while tumor nests remain immune-depleted [33]. Biologically, this pattern is consistent with an immune-excluded phenotype, in which PD-L1 expression is upregulated independently of T-cell infiltration, often due to stromal barriers or to the transforming growth factor-beta (TGF-β) pathway, a major cellular signaling system that plays a dual role in cancer —acting as both a tumor suppressor in early stages and a tumor promoter in advanced disease. TGF-β signaling [38]. Similar immune-excluded, PD-L1^+^/CD8^−^ profiles have been associated with poor prognosis and limited response to checkpoint inhibitors in both cervical and other solid tumors [36,37]. These findings support the value of incorporating spatial immune profiling to improve immune phenotyping and guide patient selection for immunotherapy.

Importantly, PD-L1 positivity was significantly associated with advanced FIGO stage and high Ki-67 index, both markers of aggressive tumor behavior. These findings reinforce the concept that PD-L1 may also reflect intrinsic tumor biology, characterized by genomic instability, increased proliferation, and immune evasion mechanisms [33,36]. Previous studies have shown similar trends, with higher PD-L1 expression correlating with worse differentiation, lymph node metastasis, and treatment resistance [7,16]. Our data contributes to this evidence by highlighting the association of PD-L1 with high-risk features in untreated invasive SCC, a group that is less frequently explored in PD-L1 studies.

In addition, in the present study, statistically significant differences were noted with other adverse features like parametrial invasion, advanced FIGO stage, lympho-vascular invasion, and the higher rate of nuclear proliferation with positive correlation, which is consistent with a more aggressive, genomically unstable proliferative disease biology.

Our research demonstrates that TME in invasive SCC of the cervix is strongly influenced by the balance between PD-L1 expression and CD8^+^ TILs. A high CD8^+^ TILs density was associated with favorable features, including a lower proliferation index or absent lympho-vascular space invasion, while PD-L1 positivity correlated with an advanced FIGO stage, lymph node metastases, parametria invasion, and proliferation rate. Importantly, PD-L1 expressions showed a significant negative correlation with CD8^+^ TIL density, highlighting their complementary prognostic value. Patients with PD-L1 positivity and low CD8^+^ TILs had significantly limited overall survival compared to patients with negative PD-L1 and abundant CD8^+^ TILs. These findings reinforce the observations made by Chen J. et al. (2020) and Tang Y. et al. (2021) [33,37], suggesting that the combined assessment of PD-L1 and CD8^+^ TILs may guide selection for immunotherapeutic strategies in cervical cancer, especially those with advanced clinical stage.

The PD-L1^+^/CD8^+^ low subgroup likely reflects an immune-silent tumor microenvironment, where PD-L1 contributes to immune evasion in the absence of sufficient cytotoxic response. These tumors may represent an immunologically “cold” phenotype, characterized by both immune suppression and exclusion. Identifying such a phenotype pre-treatment could help stratify patients with innately poor prognosis and limited likelihood of response to monotherapy checkpoint inhibition. This supports the potential value of combining PD-L1 and CD8^+^ TILs status as a dual biomarker approach, rather than relying on PD-L1 alone, for both prognosis and future immunotherapy stratification.

This study has several limitations that should be acknowledged. It was conducted at a single center, which may limit the generalizability of the findings to broader populations. The sample size was relatively small (n = 48), and the number of survival events was limited, which may affect the statistical power and robustness of multivariate analyses. HPV typing was not performed, preventing assessment of potential associations between HPV genotype and immune marker expression. Future prospective, multi-center studies with larger cohorts and integrated HPV subtyping are needed to validate and expand upon these findings.

## 5. Conclusions

This new insight into the tumoral microenvironment highlights meaningful prognostic markers and emphasizes the need for targeted immunotherapeutic strategies that leverage the presence and activity of CD8^+^ TILs and PD-L1 expression to improve clinical outcomes in cervical cancer patients. Our findings suggest that PD-L1 expression and CD8^+^ TILs density have complementary prognostic value in newly diagnosed, treatment-naive cervical squamous cell carcinoma. PD-L1 positivity was associated with more aggressive tumor features, while high CD8^+^ TIL infiltration correlated with favorable pathological characteristics and improved overall survival. Notably, the combination of PD-L1 positivity and low CD8^+^ TILs identified a subgroup of patients with poorer outcomes, supporting the relevance of dual-marker assessment in risk stratification.

These results underscore the importance of further exploring the inter-relationship between CD8^+^ TILs and PD-L1 expression with various prognostic factors to enhance understanding of their dynamics. Further validation in larger, prospective cohorts is warranted to confirm the clinical utility of combined PD-L1/CD8^+^ evaluation and to explore its potential role in guiding future therapeutic strategies.

## Figures and Tables

**Table 1:** Correlation between PD-L1 status with CD8^+^ TILs immunostain and main clinico-pathological features.

Clinico-Pathological Features		PD-L1 Negative *n* (%)	PD-L1 Positive *n* (%)	*p* Value
		15 (31, 2)	33 (68.8)	
Age (years old)	<56.5	6 (40.0)	18 (54.5)	0.350
	≥56.5	9 (60.0)	15 (45.5)	
Tumor size	<4 cm	5 (33.3)	6 (18.2)	0.283
	≥4 cm	10 (66.7)	27 (81.8)	
Parametrial invasion	No	10 (66.7)	3 (9.1)	<0.001 *
	Yes	5 (33.3)	30 (90.9)	
FIGO 2018 stage	I + II	13 (86.7)	9 (27.3)	**<0.001**
	III + IV	2 (13.3)	24 (72.7)	
Grade	G1 + G2	6 (40.0)	7 (21.2)	0.293 *
	G3	9 (60.0)	26 (78.8)	
Lympho-vascular invasion	No	11 (73.3)	7 (21.2)	**0.001**
	Yes	4 (26.7)	26 (78.8)	
TILs (%)	<10	0	10 (30.3)	**<0.001 ^#^**
	10–40	2 (13.3)	13 (39.4)	
	≥40	13 (86.7)	10 (30.3)	
CD8^+^ TILs	LowCD8^+^ TILs	4 (26.7)	19 (57.6)	**0.047**
	HighCD8^+^ TILs	11 (73.3)	14 (42.4)	
Metastasis of lymph node	No	14 (93.3)	20 (60.6)	**0.037**
	Yes	1 (6.7)	13 (39.4)	
Ki-67 index (%)	<42%	10 (6.7)	8 (24.2)	**0.009**
	≥42%	5 (33.3)	25 (75.8)	

*—Fisher test; ^#^—Chi—square “test for trend”; the bold values are statistically significant.

**Table 2:** Multivariate analysis of clinical, morphological, and immunohistochemical biomarkers for overall survival in our cohort of patients with invasive SCC.

Variable	*p*	HR (95%CI)
Age (<56.5/≥56.5 years old)	**0.007**	4.678 (1.533–14.277)
Early FIGO stage (I + II)/advanced FIGO stage (III + IV)	**0.031**	3.124 (1.109–8.796)
Tumor size (<4 cm/≥4 cm)	0.131	3.111 (0.714–13.557)
Grade of differentiation G1 + G2/G3	0.276	1.994 (0.577–6.894)
Lympho-vascular space invasion (No/Yes)	**0.009**	14.960 (1.985–112.748)
Lymph node metastases (No/Yes)	**<0.001**	5.828 (2.255–15.06)
Low CD8^+^ TILs/high CD8^+^ TILs	**0.001**	0.112 (0.032–0.390)
Negative PD-L1/positive PD-L1	**0.021**	10.730 (0.012–0.702)
Ki-67 index (<42%/≥42%)	**0.027**	4.085 (1.178–14.160)

Bold values are statistically significant.

## Data Availability

The original contributions presented in this study are included in the article; further inquiries can be directed to the corresponding author.

## Abbreviations

Each abbreviation is explained first in the text.

CIN	Cervical Intraepithelial Neoplasia
CPS	Combined Positive Score
DFS	Disease-Free Survival
FFPE	Formalin-Fixed Paraffin-Embedded
FIGO	International Federation of Gynecology and Obstetrics
H&E	Hematoxylin and Eosin
HPF	High-Power Field
HPV	Human Papillomavirus
HR	Hazard Ratio
IHC	Immunohistochemistry
LVSI	Lymphovascular Space Invasion
OS	Overall Survival
PD-1	Programmed Death-1
PD-L1	Programmed Death-Ligand 1
SCC	Squamous Cell Carcinoma
TILs	Tumor-Infiltrating Lymphocytes
TME	Tumor Microenvironment
